# Nagoya Protocol and Infectious Diseases: Hindrance or Opportunity?

**DOI:** 10.3389/fpubh.2020.00238

**Published:** 2020-06-16

**Authors:** Claire Lajaunie, Serge Morand

**Affiliations:** ^1^Inserm, LPED (Laboratoire Population Environnement Developpement), Marseille, France; ^2^Strathclyde Centre for Environmental Law and Governance (SCELG), Law School, Strathclyde University, Glasgow, United Kingdom; ^3^Centre National de la Recherche Scientifique (CNRS)—Centre de Coopération Internationale en Recherche Agronomique pour le Développement (CIRAD), Montpellier Université, Montpellier, France; ^4^Faculty of Tropical Medicine, Mahidol University, Bangkok, Thailand

**Keywords:** pathogen sharing, Nagoya protocol on access to genetic resources and the fair and equitable sharing of benefits, infectious disease, biodiversity, social equity, global health

The recent outbreak novel 2019 coronavirus (SARS-CoV-2) and the subsequent race to find its reservoir and intermediate host underline the need for swift collaborative research to be conducted all over the world. With research on infections at the animal–human interface, strong concerns have emerged in the health science community over the application of the Nagoya Protocol concerning the sharing of pathogens (and microbiota) collected from humans (1–3).

The aim of this policy-brief is not to question the principle of access and benefit sharing presented in the Nagoya Protocol (NP). Rather, our point is to determine how to help countries to implement the NP particularly on these issues and avoid that researchers operate *de facto* illegally. There is need for a science-policy dialogue among States but also at the level of the international governance regarding notably the issue of the origin of the pathogens, what the expression “utilization of genetic resources” used in the NP encompasses in this context, what could be considered as common good and what should be under the general ABS/Nagoya Protocol, to which extent specific WHO framework apply.

The NP itself and thus its implementation goes with many uncertainties, particularly when it comes to research on infectious diseases. It has been underlined regarding the biobanks and pathogens sharing (4), the specific context of eminent emergencies with a dedicated framework (such as WHO-Pandemic Influenza Preparedness Framework) (5) or the necessity of a simplified procedure associated to Nagoya Protocol implementation.

From a legal point of view the contentious issues related to the scope of the NP that have been debated during the adoption of the NP remain unclear. To reach a compromise, these thorny issues have been smoothed in a diplomatic language expressed with “constructive ambiguity” (6), but they are, in fact, unsolved.

Currently there are 123 parties to the NP and 174 Access and Benefit Sharing (ABS) National focal points. Informal ABS systems preexisted the NP, while some countries adopted legislations based on the sole provisions of the Convention on Biological Diversity after the adoption of the NP (7), but the specificity of the NP is to extend the obligation contained in one of the three objectives of the CBD, the fair and equitable sharing of benefits arising out of the utilization of genetic resources. With a growing number of parties to the Protocol, obscurities in the expression used in the NP should be uncovered and debated in light of issues linking to pathogens sharing.

We have to keep in mind that the NP is a text of international environmental law aiming at international equity and solidarity with regard to benefits arising out of the utilization of genetic resources in order “to contribute to the conservation and sustainable use of biological diversity, poverty eradication and environmental sustainability” (Preamble of the NP). Its role is notably to fight against misappropriation of natural resources.

In relation to the non-commercial public research, various gray areas still exist regarding the wording and thus the subsequent interpretation of the NP for its implementations. We notice a lack of consideration of specific issues in the NP and the subsequent Meetings of the Parties (MOPs).

## The Scope of the NP: a Broad and Multi-Layered Definition

The scope of the NP as written in the article 3 is:

“This Protocol shall apply to genetic resources within the scope of Article 15 of the Convention and to the benefits arising from the utilization of such resources. This Protocol shall also apply to traditional knowledge associated with genetic resources within the scope of the Convention and to the benefits arising from the utilization of such knowledge.”

Article 15 of the CBD about Genetic resources states:

“1. Recognizing the sovereign rights of States over their natural resources, the authority to determine access to genetic resources rests with the national governments and is subject to national legislation.
2. Each Contracting Party shall Endeavor to create conditions to facilitate access to genetic resources for environmentally sound uses by other Contracting Parties and not to impose restrictions that run counter to the objectives of this Convention (…)”.

If the question of Prior informed consent (PIC) and Mutually Agreed Terms (MAT) is widely debated and well-known, other issues have been left aside. It is the case of the utilization of genetic resources, defined very broadly by the NP as “to conduct research and development on the genetic and/or biochemical composition of genetic resources, including through the application of biotechnology as defined in Article 2 of the Convention”. As we can see, for instance the expression “research and development” has to be defined and detailed in the context of the NP as well as the expression “genetic and/or biochemical composition.” And if we were to find clarification in the article 2 of the CBD regarding biotechnology, we might be disappointed as biotechnology means “any technological application that uses biological systems, living organisms, or derivatives thereof, to make or modify products or processes for specific use.”

The article 8 of the Nagoya Protocol introduces special considerations, notably the article 8(b) which stipulates that Parties shall “Pay due regard to cases of present or imminent emergencies that threaten or damage human, animal or plant health, as determined nationally or internationally. Parties may take into consideration the need for expeditious access to genetic resources and expeditious fair and equitable sharing of benefits arising out of the use of such genetic resources (…)”.

Nevertheless, there is no detail about how to implement these special considerations. Thus, the articulation between all these various provisions supposed to define not only the genetic resources but also their utilization, may lead to different or even sometimes contradictory legal interpretations, depending on the country implementing them. The NP is implemented at the domestic level so the general provisions of the NP should avoid ambiguity as much as possible to allow an implementation in conformity with the letter of the NP.

This lack of clarity opens the door to wrong interpretations and flawed extrapolations that constitute legal nonsense, for instance considering the United States exempt from obligations under the NP because they are not party to this protocol. In this case, it is remarkable, the compliance mechanism can make non-party researchers comply. Indeed, as users, the United States are required to follow the legislation on ABS promulgated in the country providing the genetic resources (8, 9).

The provisions of the NP are written in a vague and generic way (10). This leads to difficulties when it comes to translate these international provisions into national law. Notably, it seems largely disconnected from health practices whereas it concerns genetic resources.

**Figure 1:**
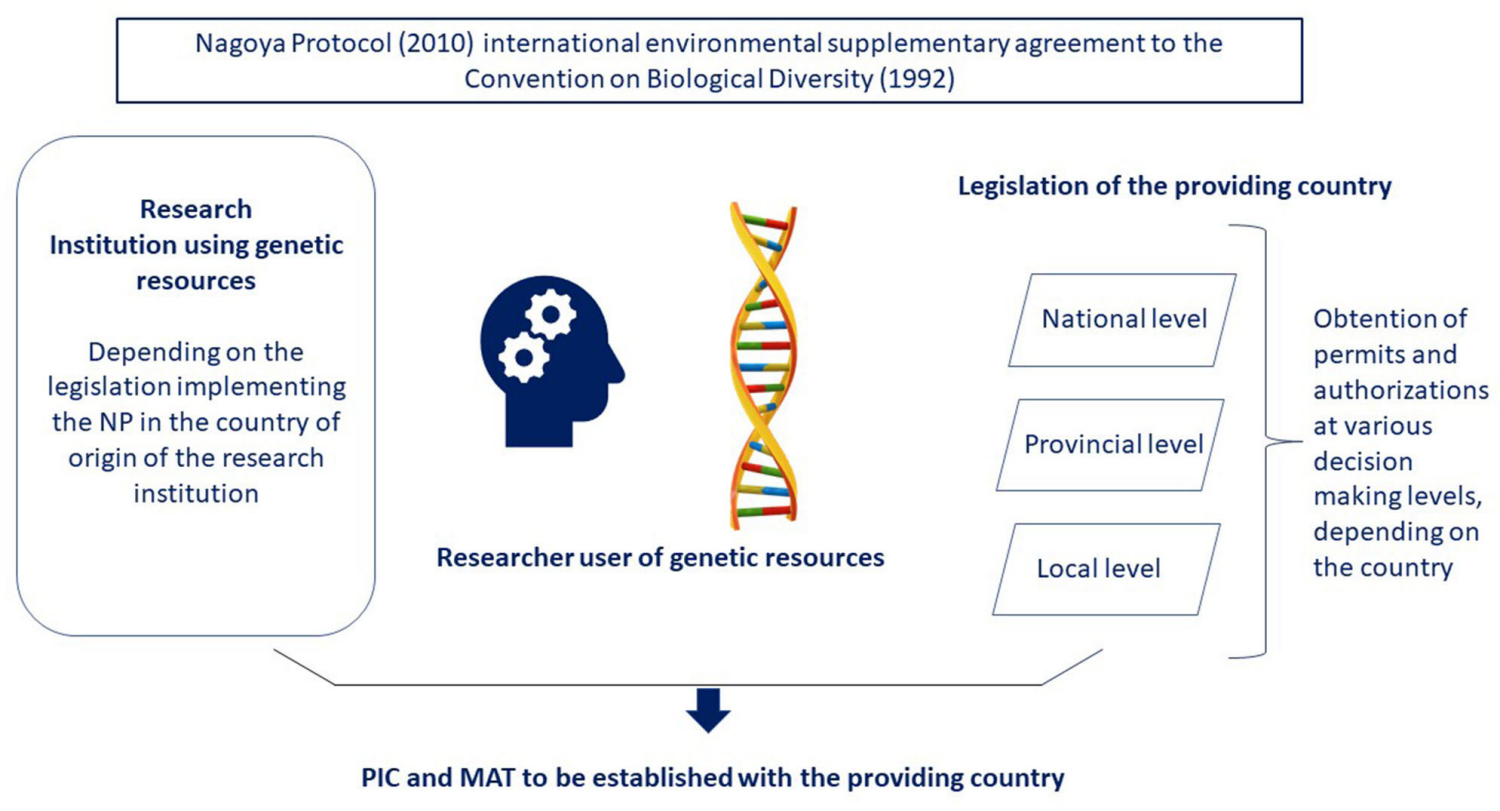
Decision making framework regarding the NP.

As a result, the implementation of the protocol into national law may differ leading to a variety of definitions of scope in ABS legislation (11). It implies a diversity of procedures among provider countries which may lead to confusion. The difficulty to access genetic resources might also result from the administrative process and the successive layers of decision-making interventions (national, provincial, local, see Figure 1) and thus the variety of permits to obtain. The implementation of the NP may sometimes circumvent the rights of local communities or indigenous people or question the acknowledgment of collective ownership within a community (12) which was not intended by the NP. This administrative burden is sometimes seen as an obstacle to an efficient research (13) and necessitates to enhance institutional capability.

## Sharing Pathogens and Microbiota for the Sake of Human Health and Biodiversity

Nevertheless, specific needs of stakeholders involved into biodiversity conservation and innovation, such as non-commercial academic research community should be considered and carefully pondered to avoid perverse effects of the implementation of the NP (14). Salient issues in relation to research in infectious diseases have not been taken much into consideration in the debates after the adoption of the NP and its implementation.

Pathogens from the wildlife are regulated under the CBD (1992) and the Convention on International Trade in Endangered Species of Wild Fauna and Flora (CITES, 1973). In turn, regarding zoonotic pathogens, the exclusion of human genetic resources (without prejudice to further consideration) from the NP at the time of its adoption could be put under scrutiny^1^. Indeed, different strains of pathogens originating from the wildlife might be found into vectors or domestic animals which in turn causes threat to human health security. It also questions the notion of consent itself as we should determine to whom it should be asked: it has to be unscrambled when it comes to domestic animal or wildlife. Another key issue arising from the NP is the absence of specific provision to help developing countries to preserve and store their own biodiversity in country (15) which could constitute a counterpart of the access granted to other countries.

The relativity of this distinction regarding the origin of the pathogens made in the NP is particularly striking when it comes to research on the microbiota. Who is the owner of the microbiota and do we have a right to microbiota? That question is raised by Ishaq et al. (16) together with the one of access to and benefit from microorganisms and its link with social equity, particularly in human. It is thus clearly an issue within the framework of the NP and it should be debated as such. The same kind of consideration applies to genetic sequences, proteomics and a large part of the knowledge linked to biodiversity, having an impact on food security, soil diversity, human, and animal health and the on the environment.

Existing specialized regimes of access and benefit sharing, but not all clearly and transparently listed, are adding complexity into the NP landscape (17). At a moment when the NP is called into question or seen as impeding scientific research (particularly in the case of health emergencies (2, 18) it appears pressing to debate and delineate clearly these various issues in relation to the implementation of the NP in order to lift the ambiguities.

Whilst the UN called the scientific community to further its work on interconnected and cross-cutting issues by sharing knowledge that inform the work of multilateral environmental agreements and environmental processes in order to advance toward a Global Pact for the Environment (19), scientists from different disciplines (such as human medicine, health anthropology and environmental law) are strongly invited to clarify and suggest changes.

Our suggestion is to translate into a practical guideline directed toward public research institutions, scientists, CBD, NP, and ABS focal points to clarify the status of pathogens. An international coordination of pathogen sharing involving the international public research and international governance in the sector of health (human and animal) and the environment is necessary to better the implementation of the NP for the sake of biodiversity and social equity.

## Author Contributions

SM and CL contributed together to the writing of the article. CL has drawn the Figure 1. All authors contributed to the article and approved the submitted version.

## Conflict of Interest

The authors declare that the research was conducted in the absence of any commercial or financial relationships that could be construed as a potential conflict of interest.

## Footnotes

^1^The possibility is stated in the decision CBD COP X Decision X/1, paragraph 5.
